# Supplementary material for: The microenvironment dictates glycocalyx construction and immune surveillance
Source: Res Sq. 2023 Aug 16:rs.3.rs-3164966. Preprint. [Version 1] doi: 10.21203/rs.3.rs-3164966/v1 (PMC10462183; doi:10.21203/rs.3.rs-3164966/v1)
Supplement: Supplement 1 [file NIHPPrs3164966v1-supplement-1.pdf]

Table S1. Lectin Microarray Information

	Description
**1. Sample: Glycan-containing sample (e.g. glycan, glycoprotein, cell lysate, cell, glycopeptide, etc.)**	
Description of Sample	Glycoproteins extracted from MCF10A cells (n = 4).
Sample preparation protocol	MCF10A cell pellets are washed with PBS supplemented with protease inhibitor cocktail. The solutions are sonicated on ice until homogenous and then ultracentrifuged to collect pellet prior to labeling.
Labeling protocol for sample detection	Samples are labelled with Alexa Fluor 555-NHS (Thermo Fisher).
Two-color reference (if used)	A pooled reference of all samples labeled with Alexa Fluor 647-NHS (Thermo Fisher).
Assay protocol	Lectin microarrays are blocked with blocking buffer (50 mM ethanolamine and 100 mM boric acid) for one hour at room temperature. Slides are rinsed once with PBST (0.01%) and once with PBS for 5 minutes each, then dried using a slide spinner. Each slide is mounted on a 24-well format hybridization cassette (Arrayit), in which each well contains a subarray. To each well, 5 µg of sample and pooled reference are added, then diluted with PBST to reach the final volume (100uL) and concentration (0.005%). Slides are incubated on an orbital shaker for one hour at room temperature in the dark. After hybridization, arrays are washed with PBST (0.01%) once for one minute, then once for five minutes. Arrays are lastly washed once with PBS for 10 minutes. Once finished, slides are removed from the cassette and briefly immersed in ultrapure water, then dried using a slide spinner.
**2. Lectin Library**	
General description of the lectin library used in the array	Lectin microarrays are generated in house.
List of lectins and/or glycan-binding proteins, their source, concentration, and buffer	Please see **Supplemental Table S2**.
Modification of lectins	N/A

3. Immobilization Surface; e.g., Microarray Slide	
Immobilization surface	Nexterion Slide H Barcoded 3D Hydrogel Coated.
Manufacturer	Schott North America.
Custom preparation of the surface	N/A
**4. Array Production**	
Description of Arrayer	Nano-Plotter 2.1 piezoelectric printer (GeSim, Germany) with cooled microwell plate holder and cooled printing deck.
Lectin deposition	Triplicates of each lectin are printed onto each subarray.
Printing conditions	Dilute lectins to the pre-determined concentrations in the print buffer (final concentration of print buffer: 1 mM monosaccharide in PBS, 5 ng/mL Atto 532; Please see Supplemental Table 1 for the concentrations of lectins). Load the mixed solution to the microplate. Before printing, check the humidity of the print chamber. The humidity should be kept around 50% throughout the entire print. Ensure both microwell plate holder and printing deck are cooled. Adjust the cooling temperature based on ambient temperature and the temperature of the cooled slide deck surface, preventing moisture building up inside the print chamber. Once printing is complete, allow the slides to dry for at least one hour.
Array layout	Each microarray contains 24 subarrays (3 columns and 8 rows). In each subarray, triplicates of a lectin are printed, with six lectins in each row. The number of columns is 18, and the row number depends on how many lectin probes are printed on the arrays (i.e. 132 lectins require 22 rows).
Quality control	The well-characterized glycoproteins glycophorin, ovalbumin, albumin, 1:1 A549:HEK293T cell lysate, human serum, fetuin, and RNase B are used for quality assurances of the printed microarrays.
**5. Detector and Data Processing**	
Instrument (scanner, flow cytometer)	Fluorescent Slide Scanner GenePix 4400A (Molecular Devices).
Instrument settings	A low resolution preview scan of the slide is performed to adjust photomultiplier tube (PMT) gain for each channel (Alexa Fluor 555: 532nm, Alexa Fluor 647: 635nm) so that the signals are not saturated and within the linear detection range. After adjusting PMT gain, slides are scanned in each channel at 5 μm resolution.
Image analysis software	GenePix Pro 7 (Molecular Devices).

Data processing and statistical analysis	Extracted data is processed for quality checks using Grubbs outlier test with $\alpha = 0.05$. Log2 values of the average signals are median-normalized over the individual subarray in each channel.
**6. Lectin Microarray Data Presentation**	
Data presentation and interpretation	Hierarchical clustering of the processed data is performed using Pearson Correlation coefficient, and volcano plots generated using R (v3.6.1) and RStudio (Build 576).
**7. Data Location**	
Data Location	**Insert doi for data or indicate where data is hosted.**

Table S2. Lectins used in microarrays

Lectin	Species/Origin	Print Conc. (µg/mL)	Rough Specificity /Inhibitory monosaccharide	Vendor/Source
AAL	*Aleuria aurantia*	2000	Fucose	Vector
ACA	*Amaranthus Caudatus*	2000	Gal-β1,3-GalNAc	EY
AIA	*Artocarpus integrifolia*	2000	β1,3-GalNAc	Vector/EY/Glycomatrix
AMA	*Allium moly*	2000	Oligo mannose	EY
Anti-B.G.A	MAb mouse IgM [HE-193]	undiluted	Blood group A antigen	Thermo Fisher
Anti-B.G.B	MAb mouse IgM [HEB-29]	undiluted	Blood group B antigen	Abcam/Thermo Fisher
Anti-E-selectin	MAb mouse IgG1 [P2H3]	undiluted	E-selectin	Thermo Fisher
Anti-Ficolin-1	MAb mouse	undiluted	Ficolin-1 antigen	Lifespan Biosciences
Anti-Ficolin-3	MAb mouse IgG1 [4H5]	undiluted	Ficolin-3 antigen	Lifespan Biosciences
Anti-Galectin-3	MAb mouse IgG1 [A3A12]	undiluted	Galectin-3 antigen	Thermo Fisher
Anti-B.G.H1	MAb mouse IgG3 [17-206]	undiluted	Blood group H1 antigen	Thermo Fisher
Anti-B.G.H2	MAb mouse IgM [A46-B/B10]	undiluted	Blood group H2 antigen	Santa Cruz Biotechnology
Anti-IgM	MAb mouse IgG1 [SA-DA4]	undiluted	IgM	Thermo Fisher
Anti-B.G. Lewis A	MAb mouse IgG1 [7LE]	undiluted	Lewis A	Abcam/Thermo Fisher

Anti-B.G. Lewis B	MAb mouse IgM [2-25LE]	undiluted	Lewis B	Abcam/Sigma
Anti-B.G. Lewis Y	MAb mouse IgM [F3]	undiluted	Lewis Y	Abcam
Anti-Lewis X [P12]	MAb mouse IgM [P12]	undiluted	Lewis X	Sigma
Anti-Mac-2bp	MAb mouse IgG1 [SP2]	undiluted	LGALS3BP	Thermo Fisher
Anti-MBL	MAb mouse IgG1 [3B6]	undiluted	Collectin-1	Abcam
Anti-P-Selectin	MAb mouse IgG1 [Psel.KO2.3]	undiluted	P-selectin	Thermo Fisher
AOL	*Aspergillus oryzae*	2000	Fucose	TCI America
ASA	*Allium sativum*	2000	Mannose	EY
BambL	*Burkholderia ambifaria*	2000	Fucose	Generated in house
BC2L-A	*Burkholderia cenocepacia*	2000	Mannose	Elicityl
Blackbean	*Blackbean crude*	2000	GalNAc	EY
BPA	*Bauhinia purpurea*	2000	β-Gal / β-GalNAc	Vector
CA	*Colchicum autumnale*	2000	Bi-antennary N-linked glycans	EY
ConA	*Canavalia ensiformis*	2000	Tri-mannose core	Vector/Thermo Fisher
CSA	*Cystisus scoparius*	2000	Terminal GalNAc	EY
DBA	*Dolichos biflorus*	2000	GalNAc	Vector
diCBM40	engineered NanI from *Clostridium perfringens*	1500	α Sialylation	Generated in house
DSA	*Datura stramonium*	2000	LacNAc	Vector/Sigma
ECA	*Erythrina cristagalli*	2000	LacNAc	Vector
GNA/GNL	*Galanthus nivalis*	2000	Oligo mannose	Vector/Sigma
GS-I	*Griffonia simplicifoia-I*	2000	α-Gal / Lac	Vector
GS-II	*Griffonia simplicifoia-II*	2000	GlcNAc	Vector
H84T	*Banana lectin*	1000	High mannose	Gift from Dr. David Markovitz
HHL	*Hippeastrum Hybrid*	2000	Oligo/High mannose	Vector/BioWorld

HPA	*Helix pomatia*	2000	Blood Group A	Sigma
LcH	*Lens culinaris*	2000	Core Fucose	Vector/Aniara Diagnostica/EY/Medicago
LEA/LEL	*Lycopersicon esculentum*	2000	GlcNAc	Vector
Lotus	*Lotus tetragonolobus*	2000	Fucose	Vector/EY
MAL-I	*Maackia amurensis-I*	2000	Sialylation/Sulfation	Vector
MAL-II	*Maackia amurensis-II*	2000	Sialylation/Sulfation	Vector
MNA-G	*Morus nigra Morniga G*	2000	GalNAc	EY
MNA-M	*Morus nigra Morniga M*	2000	Oligo mannose / Gal	EY
MPA/MPL	*Maclura pomifera*	2000	β1,3-GalNAc	Vector
NPA	*Narcissus pseudonarcissus*	2000	Oligo mannose	Vector
PHA-E	*Phaseolus vulgaris Erythroagglutinin*	2000	Bisecting GlcNAc	Vector/EY
PHA-L	*Phaseolus vulgaris Leukoagglutinin*	2000	β1,6 Branching N-Link glycans	Vector/EY
PNA	*Arachis hyogaea*	2000	Gal-β1,3-GalNAc	Vector
Recombinant Protein A	*Staphylococcus aureus*	2000	Immunoglobulins	Thermo Fisher
Recombinant Protein G	*Streptococcus* Group G	2000	Immunoglobulins	Thermo Fisher
Recombinant Protein L	*Peptostreptococcus magnus*	2000	Immunoglobulins	Thermo Fisher
PSA	*Pisum sativum*	2000	Core Fucose	Vector/Glycomatrix
PSL	*Polyporus squamosus*	2000	α2,6 sialylation	EY
PTL-II	*Psophocarpus tetragonolobus-II*	2000	α2 Fucose	Vector
RCA120	*Ricinus Communis Agglutinin I*	2000	Gal / Lac	Vector
rGRFT	*recombinant Griffithsin*	1700	High mannose	Gift from Dr. Barry O'Keefe
Ricin B Chain	*Ricinus communis*	2000	Gal	Vector
SBA	*Glycine max*	2000	LacdiNAc	Vector
SLBR-B	*Streptococcus gordonii M99*	2300	α2,6 sialylation	Generated in house
SLBR-H	*Streptococcus gordonii DL1*	2000	α2,3 sialylation	Generated in house
SLBR-N	*Streptococcus gordonii UB10712*	2000	α2,3 sialylation	Generated in house

SNA	*Sambucus nigra*	2000	α2,6 sialylation	Vector
SNA-II	*Sambucus nigra-II*	2000	α2 Fucose /oligo mannose	EY
TJA-II	*Trichosanthes japonica-II*	2000	α2 Fucose	Aniara Diagnostica
TL	*Tulipa sp.*	2000	GlcNAc	EY
UDA	*Urtica dioica*	2000	GlcNAc / Oligo mannose	EY
UEA-I	*Ulex europaaeus-I*	2000	α2 Fucose	Vector/Sigma
UEA-II	*Ulex europaaeus-II*	2000	GlcNAc	EY
VVA	*Vicia villosa*	2000	Terminal GalNAc	Vector
WFA	*Wisteria floribunda*	2000	GalNAc-β1,4	Vector
WGA	*Triticum vulgare*	2000	GlcNAc	Vector

Supplemental Figures:

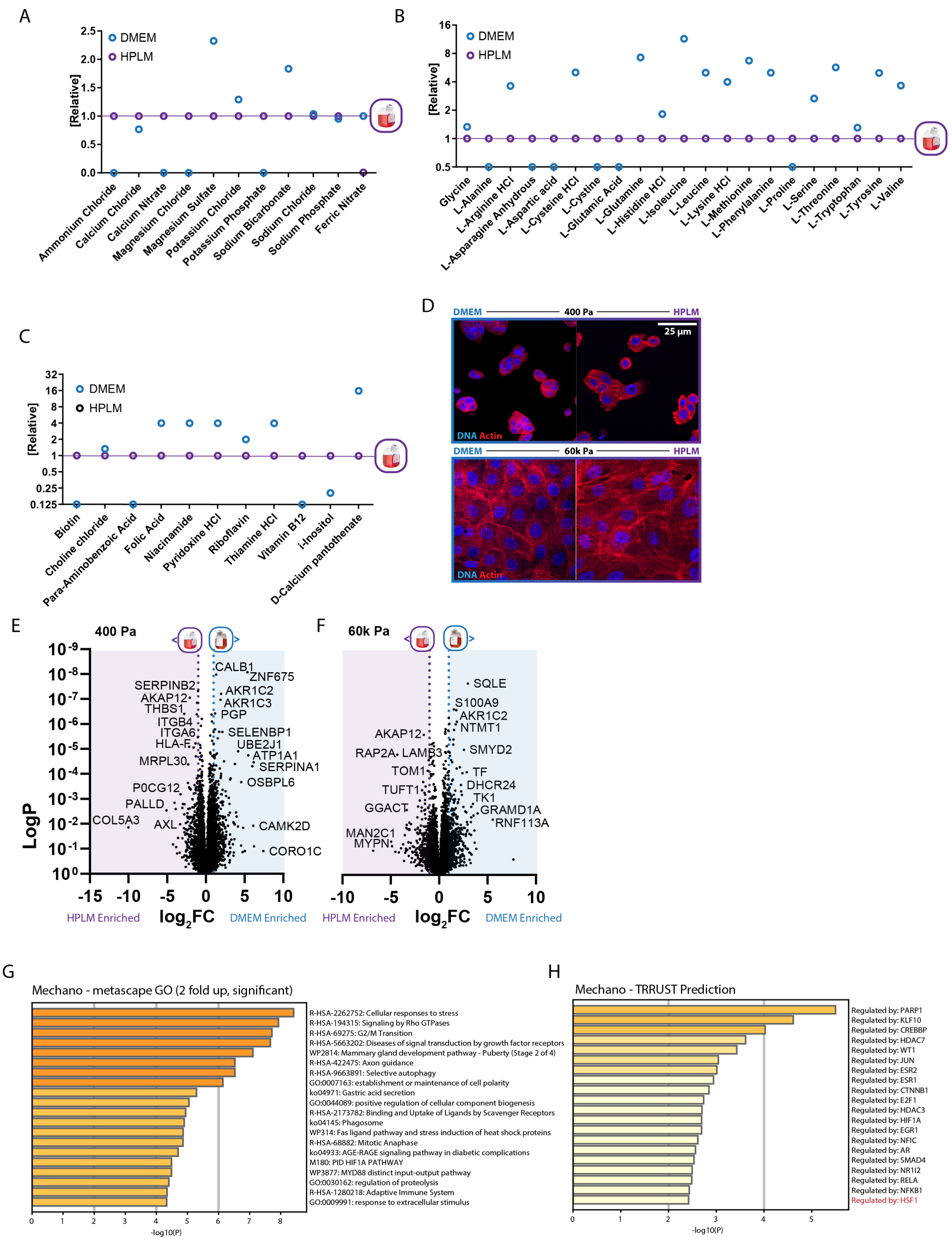

Figure S1:

A. Relative abundance of salts in HPLM or DMEM.
B. Relative abundance of amino acids in HPLM or DMEM.
C. Relative abundance of vitamins and cofactors in HPLM or DMEM.
D. Representative confocal microscopy of phalloidin (red) and DAPI (blue) staining of MCF10A cells on 400 Pa or 60k in DMEM of HPLM.
E. Volcano plot depicting relative abundance of proteins in MCF10A cells cultured on 400 Pa in HPLM vs DMEM (fold change, DMEM/HPLM).
F. Volcano plot depicting relative abundance of proteins in MCF10A cells cultured on 60k Pa in HPLM vs DMEM (fold change, DMEM/HPLM).
G. Top GO categories representative of the significantly induced (two fold and up) proteins in MCF10A cells cultured on 60k vs 400 Pa in HPLM.
H. TRRUST-based prediction of which transcription factors facilitate expression of the proteins significantly induced (two fold and up) in MCF10A cells cultured on 60k vs 400 Pa in HPLM.

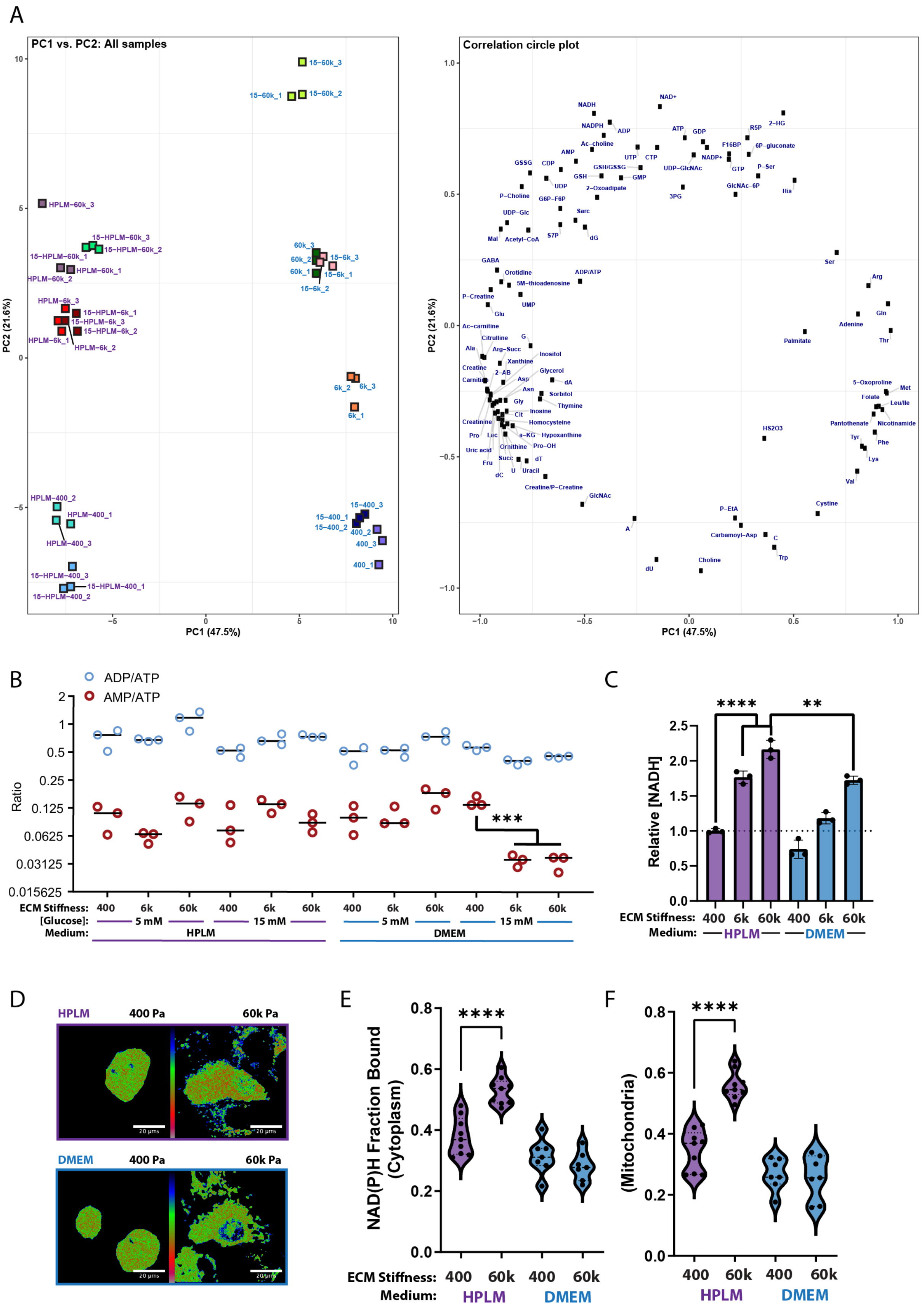
A
PC1 vs. PC2: All samples
PC1 (47.5%)
PC2 (21.6%)
15-60k_3
15-60k_1
15-60k_2
HPLM-60k_3
15-HPLM-60k_3
15-HPLM-60k_1
15-HPLM-60k_2
HPLM-60k_1
HPLM-60k_2
60k_3
60k_2
60k_1
15-6k_3
15-6k_1
15-6k_2
HPLM-6k_3
15-HPLM-6k_1
15-HPLM-6k_3
15-HPLM-6k_2
HPLM-6k_1
HPLM-6k_2
6k_2
6k_3
6k_1
HPLM-400_2
HPLM-400_1
HPLM-400_3
15-400_3
15-400_1
15-400_2
400_2
400_3
400_1
15-HPLM-400_3
15-HPLM-400_1
15-HPLM-400_2
Correlation circle plot
NAD+
NADH
NADPH
ADP
ATP
GDP
R5P
2-HG
Ac-choline
F16BP
6P-gluconate
AMP
UTP
CTP
NADP+
GSSG
CDP
GSH/GSSG
UDP-GlcNAc
GTP
P-Ser
GSH
GMP
UDP
2-Oxoadipate
GlcNAc-6P
His
P-Choline
G6P-F6P
3PG
UDP-Glc
Sarc
Mal
Acetyl-CoA
S7P
dG
GABA
Ser
Orotidine
ADP/ATP
5M-thioadenosine
Arg
P-Creatine
UMP
Gln
Glu
Adenine
Ac-carnitine
Palmitate
Thr
Citrulline
G
Ala
Arg-Succ
Inositol
Xanthine
Creatine
2-AB
Glycerol
Carnitine
Asp
dA
Asn
Sorbitol
5-Oxoproline
Met
Gly
Thymine
Folate
Leu/Ile
Inosine
Cit
Creatinine
Homocysteine
Pantothenate
Nicotinamide
Pro
Lac
a-KG
Hypoxanthine
HS2O3
Tyr
Phe
Ornithine
Pro-OH
Uric acid
Succ
dT
Lys
Fru
U
Uracil
dC
Val
Creatine/P-Creatine
GlcNAc
Cystine
P-EtA
A
Carbamoyl-Asp
C
Trp
Choline
dU
B
ADP/ATP
AMP/ATP
Ratio
2
1
0.5
0.25
0.125
0.0625
0.03125
0.015625
***
ECM Stiffness: 400 6k 60k 400 6k 60k 400 6k 60k 400 6k 60k
[Glucose]: 5 mM 15 mM 5 mM 15 mM
Medium: HPLM DMEM
C
****
**
Relative [NADH]
2.5
2.0
1.5
1.0
0.5
0.0
ECM Stiffness: 400 6k 60k 400 6k 60k
Medium: HPLM DMEM
D
HPLM 400 Pa 60k Pa
DMEM 400 Pa 60k Pa
20 µms
E
****
NAD(P)H Fraction Bound (Cytoplasm)
0.8
0.6
0.4
0.2
0.0
ECM Stiffness: 400 60k 400 60k
Medium: HPLM DMEM
F
****
(Mitochondria)
0.8
0.6
0.4
0.2
0.0
400 60k 400 60k
HPLM DMEM

Figure S2:

A. Principal Component Analysis (PCA) of intracellular metabolite levels of MCF10A cells cultured on 400, 6k, and 60k Pa in HPLM or DMEM with 5 or 15 mM glucose (indicated as 15- on plot), metabolites contributing to PC1 or 2 plotted on the right (n=3 biological replicates, associated with Fig. 2A-B)

B. AMP/ATP and ADP/ATP concentration ratios between MCF10A cells cultured on 400 Pa, 6k Pa, or 60k Pa in HPLM or DMEM with 5 or 15 mM glucose. (n=3 biological replicates, associated with Fig. 2A-B)

C. Relative abundance of NADH between MCF10A cells cultured on 400 Pa, 6k Pa, or 60k Pa in HPLM or DMEM with 5 or 15 mM glucose. (n = 3 biological replicates, associated with Fig. 2A-B)

D. Representative images of the fraction of bound NADH, calculated using the phasor approach to the fluorescence lifetime imaging (FLIM) analysis of NADH, of MCF10A cells in HPLM (top) and DMEM (bottom) media, and on 400 Pa (left) and 60 kPa (right) conditions.

E. The mean cytoplasmic fraction of bound NADH, where each point represents the average fraction bound in one field of view. (n = 7, 9 fields of view for DMEM, HPLM conditions, respectively)

F. The mean mitochondrial fraction of bound NADH, where each point represents the average fraction bound in one field of view. (n = 7, 9 fields of view for DMEM, HPLM conditions, respectively)

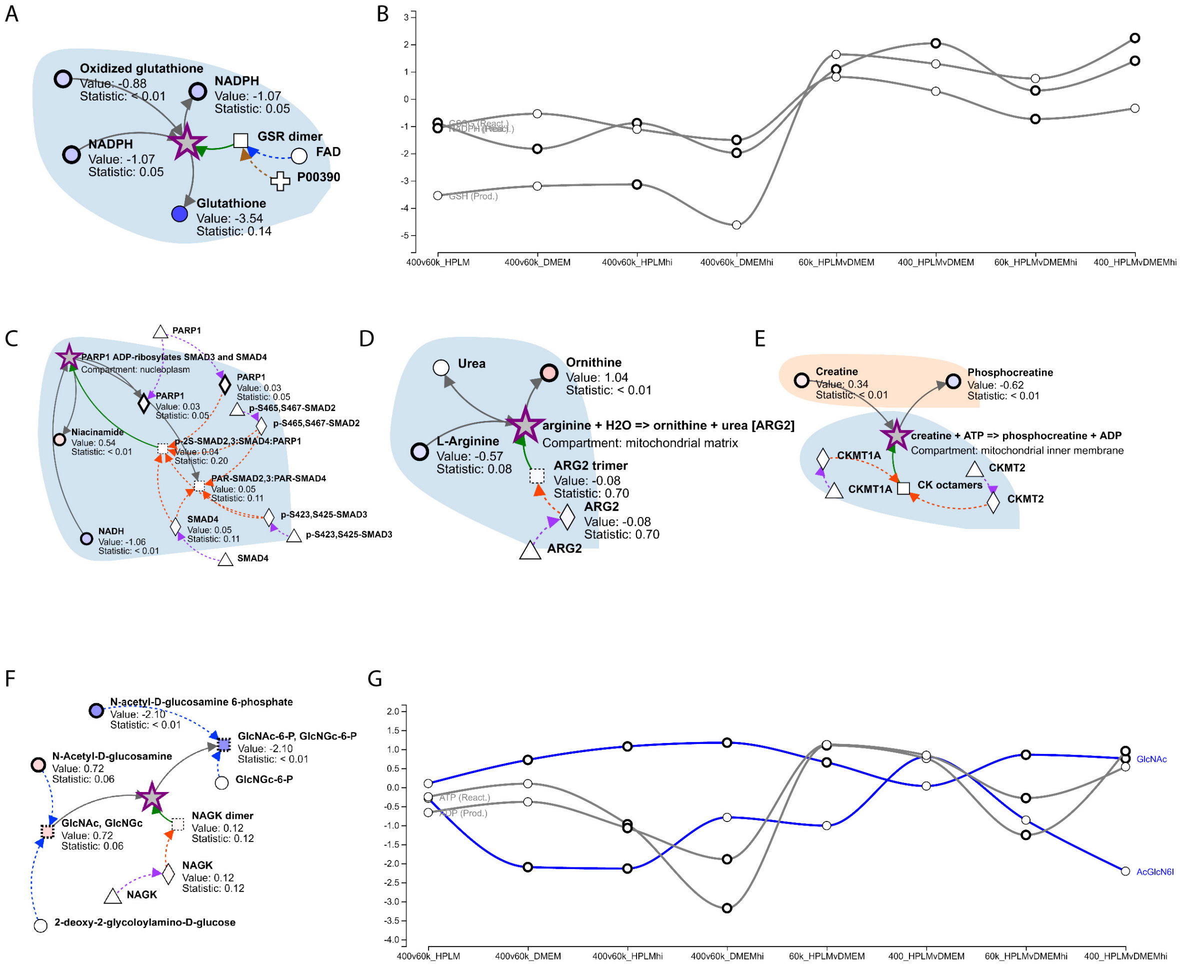

Figure S3:

A. Predicted reaction pattern to be enriched as a product of 60k Pa over 400 Pa microenvironments in HPLM more strongly than in DMEM.

B. Multiple comparison line plot of the relative abundance of products and reactants contributing to the prediction in A. Hi indicates 15 mM glucose.

C. (C-E) Predicted reaction pattern to be enriched as a product of 60k Pa over 400 Pa microenvironments, regardless of medium.

G. Predicted reaction pattern to be enriched as a product of 60k Pa over 400 Pa microenvironments in DMEM more strongly than in HPLM.

H. Multiple comparison line plot of the relative abundance of products and reactants contributing to the prediction in F. Hi indicates 15 mM glucose.

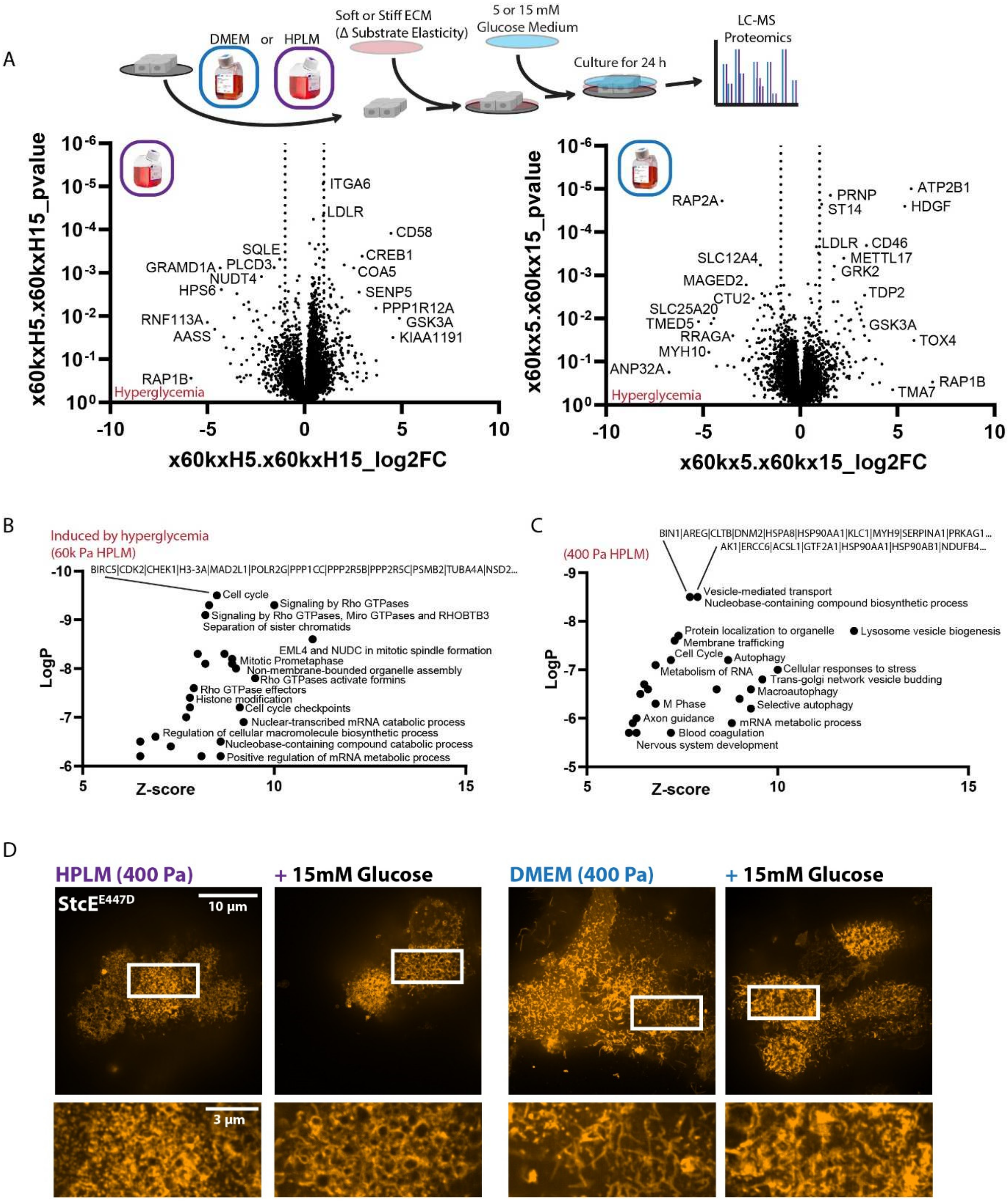
A
DMEM or HPLM
Soft or Stiff ECM
(Δ Substrate Elasticity)
5 or 15 mM
Glucose Medium
Culture for 24 h
LC-MS
Proteomics
x60kxH5.x60kxH15_pvalue
x60kxH5.x60kxH15_log2FC
ITGA6
LDLR
CD58
CREB1
COA5
SENP5
PPP1R12A
GSK3A
KIAA1191
SQLE
GRAMD1A
PLCD3
NUDT4
HPS6
RNF113A
AASS
RAP1B
Hyperglycemia
x60kx5.x60kx15_pvalue
x60kx5.x60kx15_log2FC
RAP2A
PRNP
ST14
ATP2B1
HDGF
LDLR
CD46
METTL17
GRK2
TDP2
GSK3A
TOX4
RAP1B
TMA7
SLC12A4
MAGED2
CTU2
SLC25A20
TMED5
RRAGA
MYH10
ANP32A
Hyperglycemia
B
Induced by hyperglycemia
(60k Pa HPLM)
BIRC5|CDK2|CHEK1|H3-3A|MAD2L1|POLR2G|PPP1CC|PPP2R5B|PPP2R5C|PSMB2|TUBA4A|NSD2...
Cell cycle
Signaling by Rho GTPases
Signaling by Rho GTPases, Miro GTPases and RHOBTB3
Separation of sister chromatids
EML4 and NUDC in mitotic spindle formation
Mitotic Prometaphase
Non-membrane-bounded organelle assembly
Rho GTPases activate formins
Rho GTPase effectors
Histone modification
Cell cycle checkpoints
Nuclear-transcribed mRNA catabolic process
Regulation of cellular macromolecule biosynthetic process
Nucleobase-containing compound catabolic process
Positive regulation of mRNA metabolic process
LogP
Z-score
C
(400 Pa HPLM)
BIN1|AREG|CLTB|DNM2|HSPA8|HSP90AA1|KLC1|MYH9|SERPINA1|PRKAG1...
AK1|ERCC6|ACSL1|GTF2A1|HSP90AA1|HSP90AB1|NDUFB4...
Vesicle-mediated transport
Nucleobase-containing compound biosynthetic process
Protein localization to organelle
Lysosome vesicle biogenesis
Membrane trafficking
Cell Cycle
Autophagy
Metabolism of RNA
Cellular responses to stress
Trans-golgi network vesicle budding
Macroautophagy
M Phase
Selective autophagy
Axon guidance
mRNA metabolic process
Blood coagulation
Nervous system development
LogP
Z-score
D
HPLM (400 Pa)
+ 15mM Glucose
DMEM (400 Pa)
+ 15mM Glucose
StcE^E447D
10 µm
3 µm

Figure S4:

A. Graphical representation of experimental design related to A-C and volcano plots of fold change abundance of proteins in response to hyperglycemia (enriched to left) on 60k Pa in a given medium (HPLM, left plot, purple and DMEM, right plot, blue). (n = 3 biological replicates)

B. Top GO categories representative of the significantly induced proteins in MCF10A cells cultured in hyperglycemia on 60k Pa in HPLM

C. Top GO categories representative of the significantly induced proteins in MCF10A cells cultured in hyperglycemia on 400 in HPLM.

D. Representative SoRa confocal microscopy of $StcE^{E447D}$ staining of unpermeabilized MCF10A cells cultured on 400 Pa in HPLM or DMEM with or without 15 mM glucose.

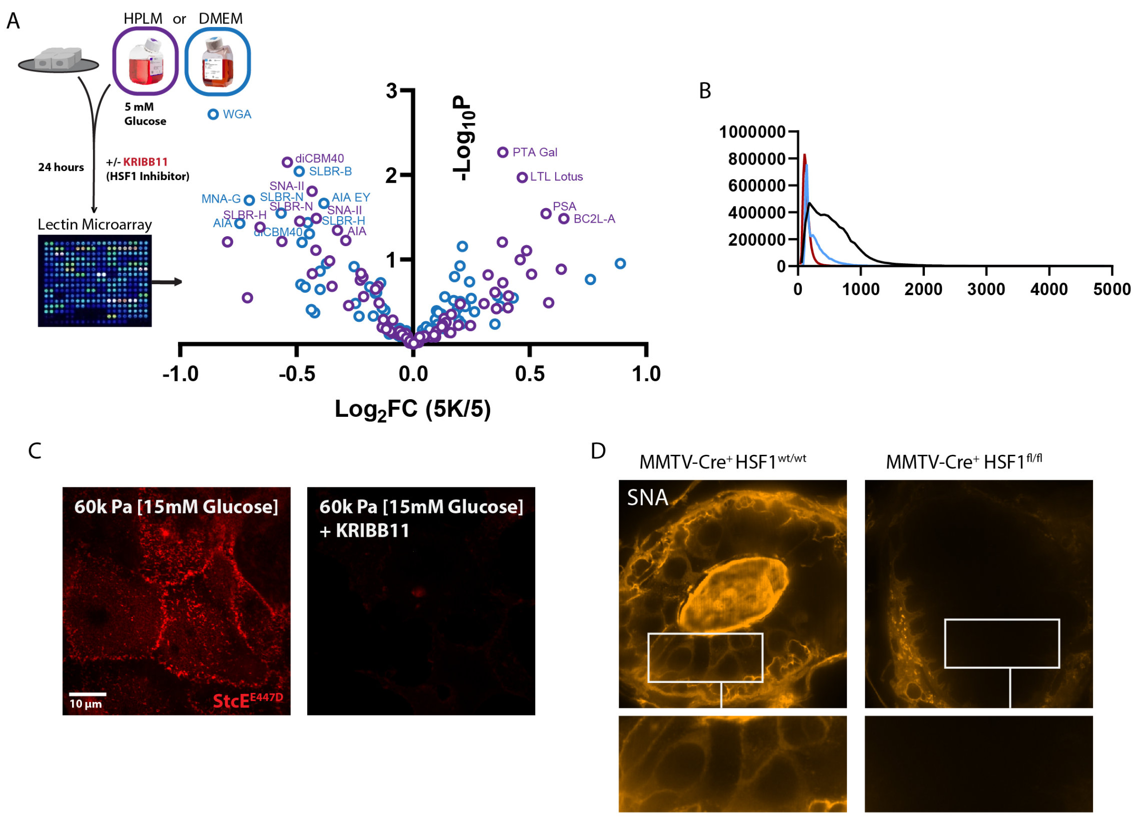

Figure S5:

A. Volcano plot depicting relative abundance of glycans from MCF10A cells cultured in HPLM with 5 mM glucose +/- KRIBB11 [2 µM], detected via lectin microarray. (n = 4 biological replicates)

B. Associated fluorescent intensity histogram for Figure 5F, KRIBB11 (red), StcE (blue), vehicle (black).

C. Representative SoRa confocal microscopy of $StcE^{E447D}$ staining of MCF10A cells on 60kPa in HPLM with 15 mM glucose +/- KRIBB11 [2 uM].

D. SNA staining associated with Figure 5G.

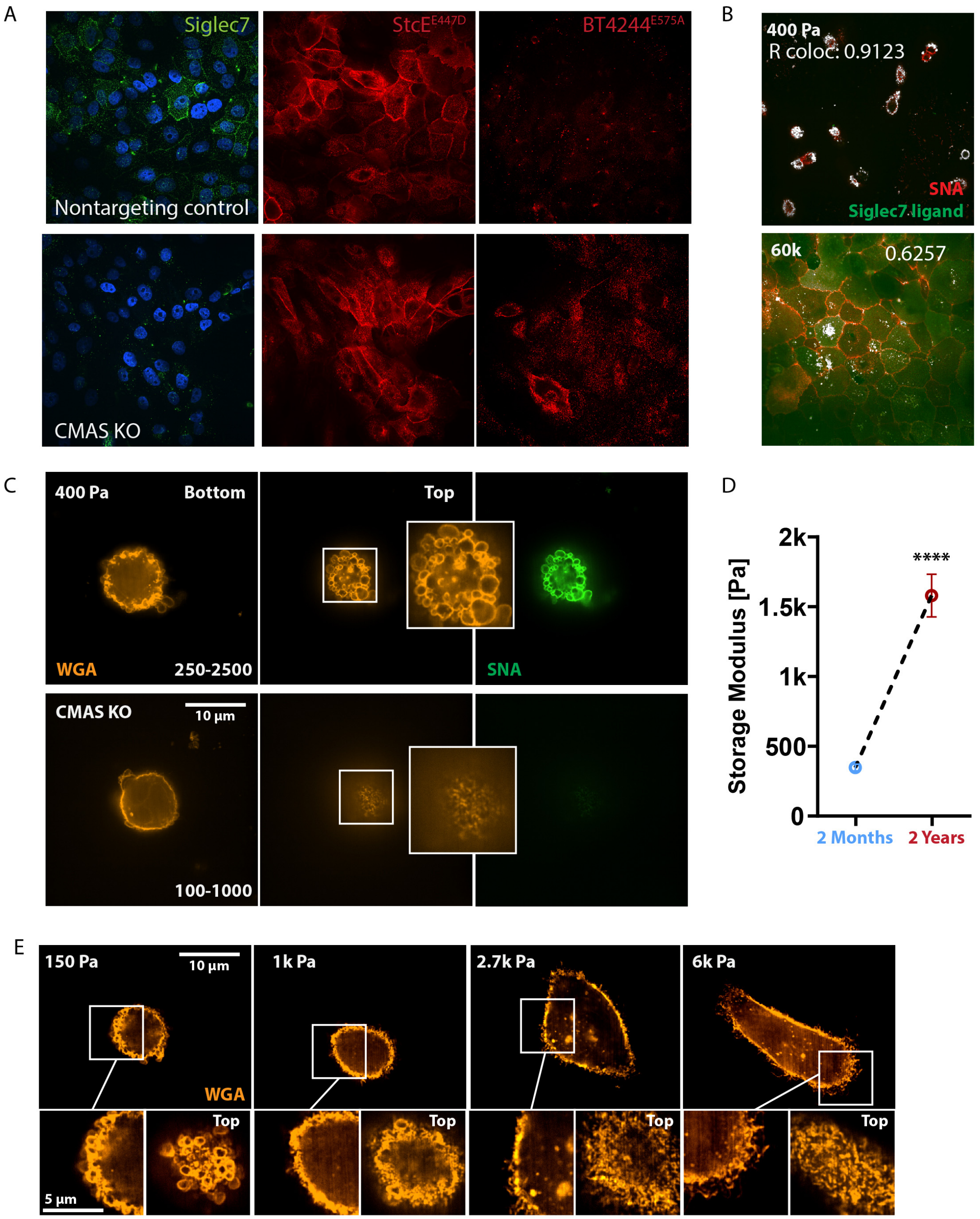
A
Siglec7
StcE^{E447D}
BT4244^{E575A}
Nontargeting control
CMAS KO
B
400 Pa
R coloc: 0.9123
SNA
Siglec7 ligand
60k
0.6257
C
400 Pa
Bottom
Top
WGA
250-2500
SNA
CMAS KO
10 µm
100-1000
D
Storage Modulus [Pa]
2k
1.5k
1k
500
0
****
2 Months
2 Years
E
150 Pa
10 µm
1k Pa
2.7k Pa
6k Pa
WGA
Top
5 µm

Figure S6:

A. Representative confocal microscopy of StcE^{E447D}(red), BT4244^{E575A} (Red), Siglec7 Fc (green), and DAPI (blue) staining of non-targeting WT controls and CMAS KO MCF10A cells in HPLM.

B. Representative confocal microscopy and colocaization (white) of Siglec7 Fc (green) and SNA (red) staining of MCF10A cells cultured on 400 Pa or 60k Pa in HPLM.

C. Representative SoRa confocal microscopy of WGA (orange) and SNA (green) staining of non-targeting WT controls and CMAS KO MCF10A cells on 400 Pa in HPLM.

D. Storage modulus of murine mammary glands from 2 month- or 2 year-old female C57BL6/J mice, measured with parallel plate rheology. (n = 5)

E. Representative SoRa confocal microscopy of WGA staining of MCF10A cells on 150 Pa, 1k Pa, 2.7k Pa, and 6k Pa in HPLM.

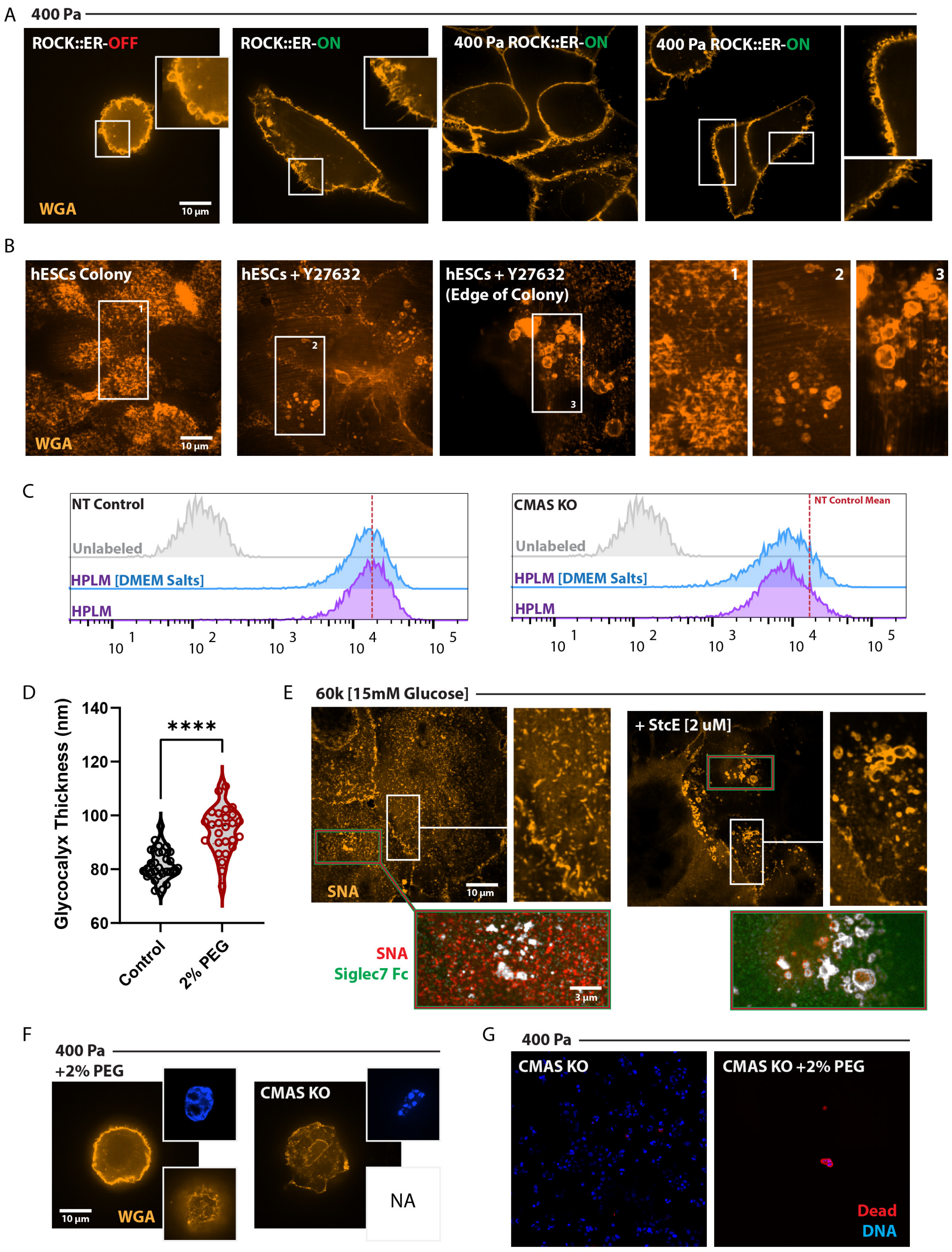
A
400 Pa
ROCK::ER-OFF
ROCK::ER-ON
400 Pa ROCK::ER-ON
400 Pa ROCK::ER-ON
WGA
10 µm
B
hESCs Colony
hESCs + Y27632
hESCs + Y27632 (Edge of Colony)
1
2
3
WGA
10 µm
C
NT Control
Unlabeled
HPLM [DMEM Salts]
HPLM
CMAS KO
NT Control Mean
Unlabeled
HPLM [DMEM Salts]
HPLM
10^1 10^2 10^3 10^4 10^5
D
Glycocalyx Thickness (nm)
140
120
100
80
60
****
Control
2% PEG
E
60k [15mM Glucose]
+ StcE [2 uM]
SNA
10 µm
SNA
Siglec7 Fc
3 µm
F
400 Pa +2% PEG
CMAS KO
10 µm
WGA
NA
G
400 Pa
CMAS KO
CMAS KO +2% PEG
Dead
DNA

Figure S7:

A. Representative confocal microscopy of WGA staining of unpermeabilized ROCK::ER MCF10A cells cultured on 400 Pa with and without 4-HT, single cells and multicellular colonies shown.

B. Representative SORA confocal microscopy of WGA staining of hESCs cultured on glass coverslips with and without 10 uM Y27632.

C. FACS analysis of WGA staining on ~ 5k non-targeting WT controls and CMAS KO MCF10A cells on in HPLM or $HPLM^{DMEM\ Salt}$.

D. SAIM-based quantification of glycocalyx thickness of MCF10A cells in HPLM with and without 2% PEG-400 for 4 hours, results are the mean ± S.D. of at least 13 cells per condition (repeated 2 separate times with similar effects).

E. Representative SoRa confocal microscopy and colocaization (white) of Siglec7 Fc (green) and SNA (orang/red) staining of MCF10A cells cultured on 60k Pa +/- StcE [2 uM] for 24 h. (+ StcE condition has fluorescent intensity enhanced 10x)

F. Representative SoRa confocal microscopy of WGA (orange) and DAPI (blue) staining of non-targeting WT controls and CMAS KO MCF10A cells on 400 Pa in HPLM with 2% PEG-400 (v/v) for 24 h.

G. Representative confocal microscopy of propidium iodide homodimer (red) and calcien-AM (white) staining of non-targeting WT controls and CMAS KO MCF10A cells on 400 Pa in HPLM with 2% PEG-400 (v/v) for 24 h.
